# Utility of the optical quality analysis system for decision-making in Nd: YAG laser posterior capsulotomy in patients with light posterior capsule opacity

**DOI:** 10.1186/s12886-020-01710-8

**Published:** 2021-01-06

**Authors:** Bo Lu, Weijie Zhu, Yu Fan, Dong Shi, Liwei Ma

**Affiliations:** 1 Department of Ophthalmology, the Fourth Affiliated Hospital of China Medical University, Eye Hospital of China Medical University, Shenyang City, 110005 Liaoning Province China; 2 Aier Excellence Eye Hospital, Central South University Aier School of Ophthalmology, Shenyang City, 110001 Liaoning Province China

**Keywords:** Posterior capsule opacification, Objective scattering index, Capsulotomy, Visual function 14 index, Visual outcomes

## Abstract

**Background:**

A prospective cohort study was performed to evaluate whether the Optical Quality Analysis System (OQAS) can serve as a valuable additional indicator for appropriate posterior capsulotomy referral.

**Methods:**

One hundred and five eyes from 96 patients undergoing capsulotomy were divided into precapsulotomy logMAR CDVA ≤0.1 group and logMAR CDVA > 0.1 group. CDVA, and the Visual Function 14 index (VF-14) score were estimated before and 1 month after capsulotomy. The objective scattering index (OSI) value was measured by using the OQAS. Posterior capsule opacification (PCO) severity was assessed with Evaluation of PCO 2000 (EPCO 2000) software.

**Results:**

In logMAR CDVA > 0.1 group, the correlations of OSI, logMAR CDVA, EPCO score and VF-14 score were very strong preoperatively. In logMAR CDVA ≤0.1 group, preoperatively, OSI was correlated with logMAR CDVA (*r* = 0.451), EPCO score (*r* = 0.789), and VF-14 score (*r* = 0.852). LogMAR CDVA has weak correlation with VF-14 score (*r* = − 0.384) and EPCO score (*r* = 0.566). VF-14 score was correlated with EPCO score (*r* = − 0.669). In the logMAR CDVA ≤0.1 group, there was no significant difference in logMAR CDVA between precapsulotomy and postcapsulotomy (*P* > 0.05). In the two groups, all the other optical quality parameters were significantly improved after capsulotomy (*P* < 0.05). In logMAR CDVA > 0.1 group, the area under the curve of the ROC of the OSI was 0.996 (*P* = 0.000). In logMAR CDVA ≤0.1 group, the area under the curve of the ROC of the OSI was 0.943 (*P* = 0.000).

**Conclusions:**

The OSI was useful for evaluating of PCO and prediction of beneficial capsulotomy. Especially for patients with slight PCO and better visual acuity, OSI is more valuable than CDVA and completely objective examination.

**Trial registration:**

The study protocol was registered at the Chinese Clinical Trial Registry. Register: ChiCTR1800018842 (Registered Date: October 13th, 2018).

## Background

Cataract surgery is the most common surgical procedure performed by ophthalmologists [1]. Posterior capsule opacification (PCO) is the most common delayed complication of cataract surgery [2]. The incidence of PCO was reported to be 20.7% at 2 years and 28.5% at 5 years after cataract surgery [3]. The incidence of the disease among children could even raise to 100% [4].

Neodymium: yttrium-aluminum-garnet (Nd:YAG) laser posterior capsulotomy is accepted as a standard treatment for PCO [5]. Although this procedure has been found to be easy, safe and effective, it may damage the intraocular lenses(IOL), increase intraocular pressure, lead to retinal hemorrhage, vitreous prolapse, hyphema, cystoid macular edema, retinal detachment, IOL dislocation or exacerbation of endophthalmitis [6–9].

The decision to perform laser capsulotomy was based on the percentage of the opacification area and VA [10], but there is no consensus as to the exact time at which this treatment should be carried out. Especially in the case of slight PCO, when the VA has not declined, it is still controversial whether the visual function would be improved after capsulotomy [11, 12].

The Optical Quality Analysis System (OQAS, Visiometrics, Terrassa, Spain) is based on the double-pass technique and developed to evaluate vision quality objectively [13]. The OQAS allows an objective assessment of intraocular scattering [14]. OQAS is widely used to make an objective evaluation of the grading of lens opacity, enable monitoring of cataract progression and aid in predicting optimal timing for cataract surgery [15, 16]. This study evaluated whether OQAS can serve as a valuable additional indicator for appropriate posterior capsulotomy referral, to be used as an objective guide.

## Methods

### Participants

This study was approved by the Ethical Committee of the Fourth Affiliated Hospital of China Medical University. Written informed consent was obtained from each participant. The study was registered at www.chictr.org.cn (no. ChiCTR1800018842).

Ninety-six patients (105 eyes) were enrolled in this study, collected from the Fourth Affiliated Hospital of China Medical University, diagnosed as PCO and had been undergone Nd: YAG laser posterior capsulotomy. The laser procedure was performed by the same experienced doctor(Lu B.). The diameter of capsulotomy was 4 mm. All of the patients had previous uneventful small incision phacoemulsification surgery with aspherical monofocal IOL implantation. All of the patients had varying degrees of visual disturbances, such as visual loss, photophobia or slightly blurred vision. Exclusion criteria were severe dry eyes, corneal diseases, glaucoma, retinal or macular pathologies, diabetes mellitus, the post-capsulotomy CDVA > 0.3 (logMAR acuity) and nature pupil diameter less than 4 mm. The patients were classified according to the precapsulotomy corrected distance visual acuity (CDVA) as the group of logMAR CDVA ≤0.1 and the group of logMAR CDVA > 0.1.

### Study design and analyses

In the study population, the parameters were obtained twice: before and 1 month after capsulotomy. The parameters included detailed slit lamp examination, intraocular pressure, optical coherence tomography, CDVA and the Chinese version of Visual Function 14(VF-14)index. The retinal image, degree of haze inside the eye, and condition of visual function were analyzed in terms of objective scattering index (OSI), modulation transfer function (MTF) cut-off value, Strehl ratio (SR), predicted visual acuity (PVA) 100%, PVA20%, and PVA9% measured with the OQAS II by using the double-pass technique [17].

The CDVA was measured with a rear-lighted Early Treatment Diabetic Retinopathy Study (ETDRS) chart on a logMAR scale, according to the modified Early Treatment Diabetic Retinopathy Study protocol [18].

**Fig. 1:**
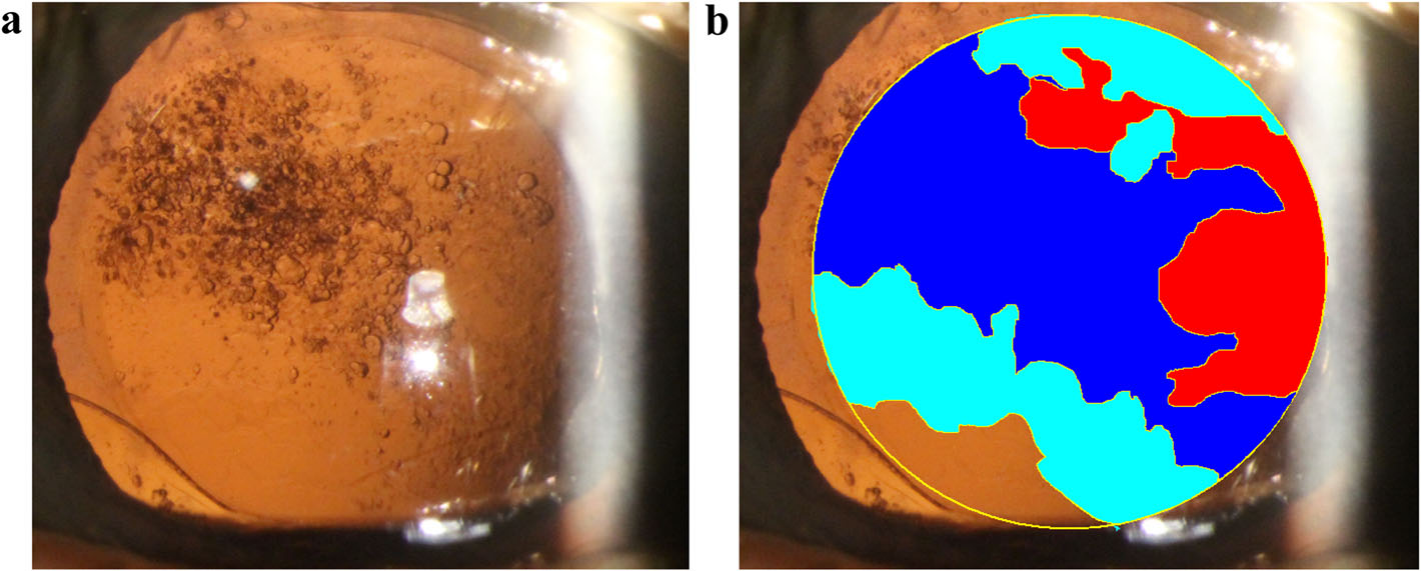
The images of the PCO evaluation using EPCO 2000 software. **a** Native image. **b** Evaluated image

For PCO severity assessment, retroillumination images of PCO were analyzed with Evaluation of PCO 2000 (EPCO 2000) software [19]. In this study, the optic area of IOL was chosen as the analysis range, and then the examiner contoured the opaque area recorded on the photograph with a mouse and graded it as 0–4 according to the density (the grading was determined by comparing pictures with instructions). There were five density categories with discrete values ranging from 0 (no opacification) to 4 (severe opacification). Fill each block with different representative colors (Fig. 1). The total PCO value was obtained by multiplying the turbidity level by the percentage of pixels occupied by the turbidity in the analysis range. EPCO score = 1 × area1 + 2 × area2 + 3 × area3 + 4 × area4.

Under the condition of the natural pupil, visual quality was examined by a clinical double-pass instrument (OQAS II, Visiometrics S.L. Tarrasa, Spain) by the same operator according to the standard operating protocol. All measurements were carried out under the 4 mm artificial pupil setting by the instrument to ensure consistency. In order to ensure that the natural pupil of the subjects was greater than 4 mm [20] during the whole measurement process, the measurements were carried out in a dark chamber to remove the refractive drift caused by further mydriasis. Before each data acquisition, the patients were instructed to blink, and the examiner collected parameters in the shortest time to avoid the influence of tear film on the accuracy of the data. All double-pass images were acquired with full correction of refractive errors. The spherical error was corrected by the OQAS automatically, which ranges from -8D to +6D. Astigmatism beyond ±0.50D was compensated by external insertion method. Three consecutive measurements were performed by the same examiner. The average of three measurements of each parameter was used for comparative analysis.

The double-pass (DP) method is an objective technique based on recording images of a point-source object after reflection on the retina and a double passage through the ocular media [21]. The MTF, OSI, SR, PVA100%, PVA20%, and PVA9% were all measured using the OQAS II. The OSI quantifies the magnitude of the intraocular scattering caused by the loss of transparency in ocular structures, such as corneal haze, cataract, and vitreous opacities [22, 23]. This index is defined as the ratio between the integrated light in the periphery and the surroundings of the central peak of the double-pass image [23]. The OSI scale ranges from 0 (no scatter) to 25 (maximum scatter). The MTF cut-off value is the frequency at which the MTF reaches a value of 0.01. It refers to the frequency up to which the eye can focus an object on the retina with a significant 1% contrast [24]. The PVAs (100, 20, and 9%) are normalized values of three spatial frequencies, which correspond to MTF values that describe the optical quality of the eye for three contrast conditions [24]. The SR is the ratio of the central maximum of the illuminance of the point spread function (PSF) in the aberrated eye to the central maximum that would be found in a corresponding aberration-free system [24].

Our research used the Chinese version of the VF-14 index to evaluate patient’s subjective visual function and quality of life. The VF-14 is a measure of perceived visual function based on 14 everyday activities that may be affected by cataracts. No questionnaire specifically assessing visual disabilities for PCO has been published, but the VF-14 has been validated in patients with other ocular diseases [25, 26]. Each participant completed the questionnaire under the guidance of the same ophthalmologist. All participants determined whether the 14 visual-related activities were affected or not due to visual impairment. If affected, the difficulty of completing the activity was graded (mild, moderate, severe and impossible). If the item was not applicable or the subject was unable to complete due to other non-visual factors, it was deleted and not used for scoring. The higher the score, the lighter the visual impairment [5].

### Statistical analysis

All analyses were performed using IBM SPSS Statistics 25.0. All data were expressed as the mean ± standard deviation. Wilcoxon signed-rank test was applied to the preoperative and postoperative comparisons. Bivariate correlation models and Spearman correlation coefficients were used to analyze the relationship between variables. Receiver operating characteristic (ROC) curve analysis was used to evaluate the OSI cut-off value. Significant differences were recorded when the *P*-value was smaller than 0.05.

## Results

Ninety-six patients (105 eyes) were enrolled in this study, ranging in age from 51 to 83 years, with an average age of 64.2 ± 11.08 years. PCO occurred 0.5–3.5 years after phacoemulsification combined with intraocular lens implantation, with an average of 1.57 ± 1.03 years.

**Fig. 2:**
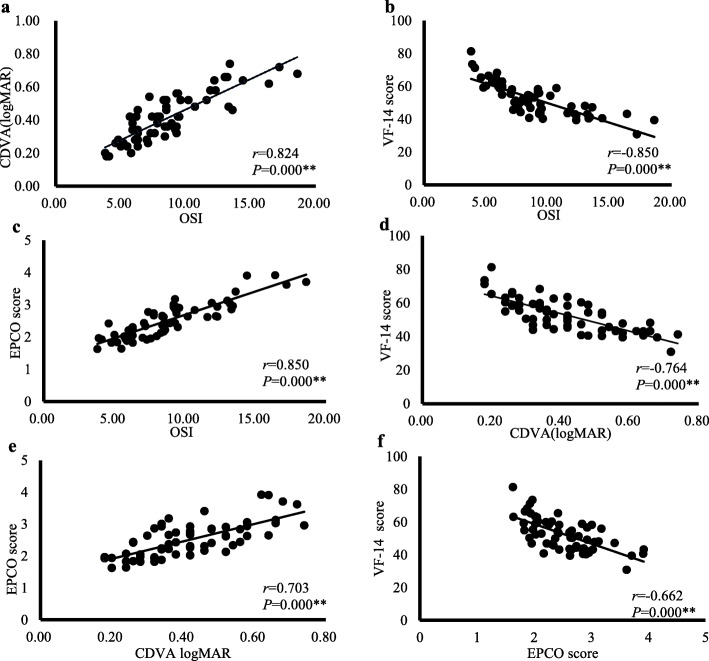
The correlations of visual function parameters in logMAR CDVA > 0.1 group preoperatively. Correlation of OSI with logMAR CDVA (**a**), VF-14 score (**b**), and EPCO score (**c**). Correlation of logMAR CDVA with VF-14 score (**d**) and EPCO score (**e**). Correlation of VF-14 score and EPCO score (**f**). *r* Pearson’s correlation coefficient; **P < 0.01

Before the laser capsulotomy, as Fig. 2 shows, in precapsulotomy logMAR CDVA > 0.1 group, OSI was significantly correlated with logMAR CDVA (*r* = 0.824, *P* = 0.000) (Fig. 2a), VF-14 score (*r* = − 0.850, *P* = 0.000) (Fig. 2b), and EPCO score (*r* = 0.850, *P* = 0.000) (Fig. 2c). LogMAR CDVA was significantly correlated with VF-14 score (*r* = − 0.764, *P* = 0.000) (Fig. 2d) and EPCO score (*r* = 0.703, *P* = 0.000) (Fig. 2e). VF-14 score and EPCO score were significantly correlated (*r* = − 0.662, *P* = 0.000) (Fig. 2f), where the correlation between OSI and VF-14 score was the strongest.

**Fig. 3:**
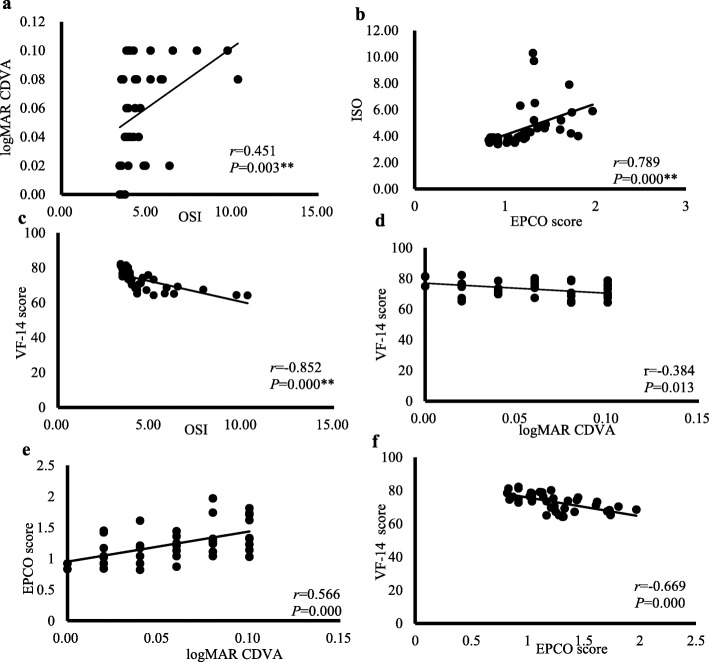
The correlations of visual function parameters in logMAR CDVA ≤0.1 group preoperatively. Correlation of OSI with logMAR CDVA (**a**), EPCO score (**b**), and VF-14 score (**c**). Correlation of logMAR CDVA with VF-14 score (**d**) and EPCO score (**e**). Correlation of VF-14 score and EPCO score (**f**). *r* Pearson’s correlation coefficient; *P < 0.05, **P < 0.01

Before the laser capsulotomy, as Fig. 3 shows, in precapsulotomy logMAR CDVA ≤0.1 group, OSI was correlated with logMAR CDVA (*r* = 0.451, *P* = 0.003) (Fig. 3a), EPCO score (*r* = 0.789, *P* = 0.000) (Fig. 3b) and VF-14 score (*r* = − 0.852, *P* = 0.000) (Fig. 3c). LogMAR CDVA has low correlation with VF-14 score (*r* = − 0.384, *P* = 0.013) (Fig. 3d) and EPCO score (*r* = 0.566, *P* = 0.000) (Fig. 3e). VF-14 score was correlated with EPCO score (*r* = − 0.669, *P* = 0.000) (Fig. 3f). The correlation between OSI and VF-14 score was the strongest, and the correlation between logMAR CDVA and VF-14 score was the lowest.

In this study, 64 eyes had logMAR CDVA > 0.1 before the operation. As shown in Table 1, 1 month after capsulotomy, subjective indexes logMAR CDVA and VF-14 score was significantly improved (logMAR CDVA, *P* = 0.000; VF-14 score, *P* = 0.000), and objective indexes OSI, MTF-cut off, SR, PVA100%, PVA20%, PVA9% were also significantly improved, compared with precapsulotomy (OSI, *P* = 0.000; MTF-cut off, *P* = 0.000; SR, *P* = 0.000; PVA100%, *P* = 0.000; PVA20%, *P* = 0.000; PVA9%, *P* = 0.000).

**Table 1:** Visual function parameters of logMAR CDVA > 0.1 group before and 1 m after laser capsulotomy

Characteristic	Precapsulotomy	Postcapsulotomy	*P* value
logMAR CDVA	0.42 ± 0.14	0.14 ± 0.09	0.000 **
OSI	8.78 ± 3.23	2.12 ± 1.37	0.000 **
VF-14 score	52.75 ± 9.66	88.43 ± 9.57	0.000 **
MTF cut off	7.73 ± 2.64	29.89 ± 9.36	0.000 **
SR	0.05 ± 0.02	0.16 ± 0.05	0.000 **
PVA 100%	0.31 ± 0.08	0.98 ± 0.11	0.000 **
PVA 20%	0.17 ± 0.03	0.48 ± 0.13	0.000 **
PVA 9%	0.09 ± 0.02	0.31 ± 0.09	0.000**

Data were expressed as mean ± standard deviation (SD). Values were tested with Wilcoxon signed-rank test. *n* = 64, ** *P* value < 0.01

In this study, the precapsulotomy logMAR CDVA of 41 eyes were less than or equal to 0.1, and the average logMAR visual acuity was 0.06 ± 0.03. All these patients complained visual disturbance symptoms of varying degrees. The precapsulotomy average VF-14 score of these patients was 73.68 ± 5.21. After mydriasis, detailed fundus examination and OCT examination were performed to exclude retinal diseases, and then laser posterior capsulotomy was performed. As shown in Table 2, there was no significant difference in logMAR CDVA between precapsulotomy and postcapsulotomy (*P* > 0.05), VF-14 scores were significantly improved compared with precapsulotomy (*P* = 0.000). The objective indexes of OSI, MTF-cut off, SR, PVA100%, PVA20%, PVA9% were significantly improved compared with precapsulotomy (OSI, *P* = 0.000; MTF-cut off, *P* = 0.000; SR, *P* = 0.000; PVA100%, *P* = 0.000; PVA20%, *P* = 0.000; PVA9%, *P* = 0.000).

**Table 2:** Visual function parameters of logMAR CDVA ≤0.1 group before and 1 m after laser capsulotomy

Characteristic	Precapsulotomy	Postcapsulotomy	*P* value
logMAR CDVA	0.06 ± 0.03	0.04 ± 0.02	0.12
OSI	4.63 ± 1.54	1.97 ± 1.37	0.000 **
VF-14 score	73.68 ± 5.21	91.35 ± 10.28	0.000 **
MTF cut off	15.23 ± 8.21	31.47 ± 11.32	0.000 **
SR	0.07 ± 0.02	0.16 ± 0.07	0.000 **
PVA 100%	0.43 ± 0.29	1.08 ± 0.13	0.000 **
PVA 20%	0.28 ± 0.11	0.48 ± 0.15	0.000 **
PVA 9%	0.14 ± 0.04	0.35 ± 0.13	0.000 **

Data were expressed as mean ± standard deviation (SD). Values were tested with Wilcoxon signed-rank test. *n* = 41, ** *P* value < 0.01

**Fig. 4:**
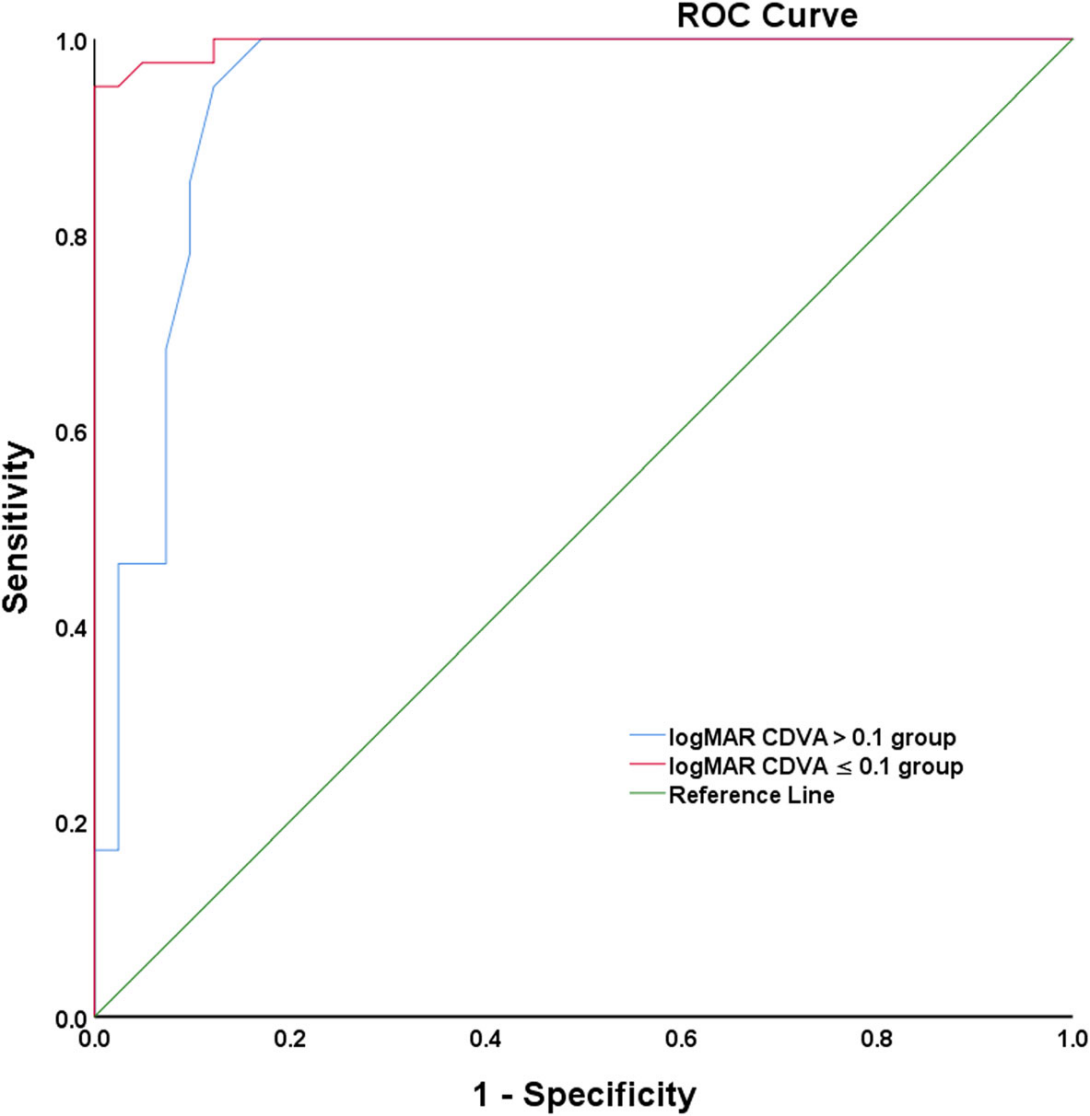
The ROC curves for the OSI in logMAR CDVA > 0.1 group and logMAR CDVA ≤0.1 group

According to the ROC curve analysis (Fig. 4), in precapsulotomy logMAR CDVA > 0.1 group, the area under the curve (AUC) of the OSI was 0.996 (*P* = 0.000). In precapsulotomy logMAR CDVA ≤0.1 group, the AUC of the OSI was 0.943 (*P* = 0.000), suggesting that the OSI had high sensitivity and accuracy in the both groups.

## Discussion

At present, the clinical evaluation of laser capsulotomy indications is still mainly based on slit lamp examination and the degree of visual impairment [10]. Nowadays, visual impairment defined by VA alone is not enough to reflect the subjective disability [27]. The slit lamp evaluation method depends more on the clinical experience of the examiner, which is easy to cause bias. In this study, the logMAR CDVA of 39% of the studied eyes was less than or equal to 0.1 before surgery. For these patients, precapsulotomy corrected visual acuity remained at a very good level; doctors tended to ignore the effect of PCO on the visual function of these patients. Therefore, it is still a difficult problem for clinicians to comprehensively evaluate the damage of PCO on the visual function of these patients and the choice of operation time. In this study, PCO severity, CDVA, objective visual function index OSI and subjective visual quality index VF-14 score were used to evaluate PCO, and the effectiveness of various methods was discussed. This study found that OSI is a good objective index for evaluating visual function changes caused by PCO. The high correlation between OSI and subjective visual quality index VF-14 was reported for the first time. The combination of OSI and subjective visual quality index VF-14 can effectively guide surgical decision-making for early PCO.

This study found that in patients with good baseline vision, a single VA was not enough to reflect PCO’s impairment on the visual quality of patients. Because some PCO patients reported obvious visual interference, but not accompanied by a decline in VA, the surgical decision-making of these patients is more complicated, so this study divided the patients into logMAR CDVA > 0.1 group and logMAR CDVA ≤0.1 group according to the precapsulotomy VA of PCO patients. This study found that precapsulotomy VA was highly correlated with objective visual quality index (OSI), subjective visual quality index (VF-14), and EPCO score in patients with apparent visual impairment preoperatively. In contrast, in patients with light visual impairment, VA has a low correlation with OSI, VF-14 and EPCO score. Nowadays, acuity is associated with the quality of daily life. However, it has been suggested that other parameters, such as stereopsis, visual field or contrast sensitivity, are more critical than acuity for functional tasks, especially since many cataract patients have relatively good acuity at baseline [28]. Previous research shows that VA is limited as a predictor of satisfaction with vision and of visual function (using the VF-14) [29, 30]. Van Bree MC et al. also found that the relation between PCO severity and logMAR is curvilinear, so the functional visual effect of slight PCO cannot be objectified by CDVA testing [31].

The main finding of this study for ophthalmic practice is that OSI can be used as a meaningful indicator of the capsulotomy.

Zhang H et al. reported that the presence of PCO reduced the patient’s visual quality and was manifested as an increase in image scattering index in the double-pass system [32]; there was a close correlation between the decline of VA and the increase of OSI [32], while van Bree MC et al. reported the relation between VA and stray light was limited in PCO [31]. This study demonstrated a good correlation between CDVA and OSI in patients with seriously visual impairment induced by PCO, but the correlation between CDVA and OSI was lower in patients with better baseline visual acuity. Because distinct optical processes cause stray light and VA impairment. Only when these two optical processes are damaged at the same time, VA and stray light may change simultaneously.

Light-scatter is more sensitive to slight PCO than contrast sensitivity and visual acuity [33]. The relation between PCO severity and straylight is linear [31], the OSI was also correlated with objective lens nuclear density in eyes with age-related nuclear cataract [15]. Nuclear, cortical, and posterior subcapsular cataracts led to a decrease in vision and an increase in the OSI [34]. Our study also found that OSI was highly positively correlated with PCO severity in all patients, and higher than the correlation between CDVA and PCO severity. The visual acuity of patients with light PCO is normal, but OSI has increased, and VF-14 score has begun to decline, which indicates that OSI reflects the changes of visual function caused by PCO earlier and more sensitive than vision. The influence of PCO position and shape on visual function has reached a basic consensus [31, 35]. In early PCO, the light intensity is slightly reduced due to slight opacity and incomplete occlusion of the optic axis, so the visual acuity remains unchanged. However, the uneven posterior capsular opacity causes light scattering in the eyes, which leads to an increase of OSI. In fibrotic PCO, turbid cells mainly originate from cubic epithelial cells of the anterior capsule. The slender white linear fibroid changes mostly begin at the edge of the IOL, which mainly causes the slight folding and thickening of the posterior capsule [36, 37]. Therefore, early fibrotic PCO only slightly reduces the illumination, which has a slight effect on central vision, but the scattering of light in the thickened area of posterior capsule fold results in the glare; as a consequence, fibrosis-type PCO may affect stray light to a larger extent than VA [37]. In Elschnig’s pearls, the enlarged cystic cells of pearl turbidity originate from the equatorial epithelial cells, which proliferate actively and migrate to the entire posterior capsule surface [38]. Elschnig’s pearl bodies act as refractors to scatter light into the eyes, so the increase of OSI is more obvious. The correspondence between OSI and PCO severity suggests the applicability of using these objective parameters to assess the severity of PCO and to make decisions about surgery.

We firstly proved the high correlation between OSI and VF-14 score in PCO. In the research, 39%(41 /105) eyes have good CDVA, but the subjective visual disturbance is noticeable. To complement objective VA information, it is now accepted that evaluating the impact of a disease on quality of life using a patient-reported outcome is important for medical interventions, therapeutic decisions, or outcomes research. De Juan-Marcos et al. found that the VF-14 scores of patients correlate more strongly with their satisfaction with vision than VA, and VF-14 is more sensitive to functional disability caused by PCO and to capsulotomy improvements, however, improvement in VA is slightly correlated with gains in quality of life [5]. In our current study, regardless of baseline vision, the correspondence between OSI and VF-14 score is very high, while in patients with better baseline vision, the correlation between CDVA and VF-14 is poor. Many studies indicated that OSI could quantitatively measure the intraocular scattering, which could be used as an objective index to reflect optical performance [39, 40]. The double-pass image was affected by both the forward and the backward scattering, produced in the first and the second pass of the light through the lens, the analysis of the energy distribution on this image revealed the contribution of the light scattering which really impaired visual performance [39]. The good consistency between the objective visual function index OSI and the subjective visual function index VF-14 score suggested that OSI can objectively verify the visual interference caused by PCO in patients. OSI is of great significance in early surgical decision-making of PCO, serving as an indicator for capsulotomy referral, distinguishing between early, beneficial capsulotomy and early, unbeneficial capsulotomy.

Nd: YAG laser capsulotomy is accepted as the standard treatment for PCO and has been found to be safe and effective [10]. Improvement in visual acuity after Nd: YAG laser capsulotomy in patients with significant PCO has been well documented [10, 41]. Improvements in glare and contrast sensitivity may also be an important outcome for many patients [10, 42]. In our study, all patients had OSI values greater than or equal to 3, regardless of the baseline VA. In the 64 eyes suffered from obvious visual impairment caused by PCO, the visual acuity and various visual function indexes improved significantly after the operation. In the 41 eyes with good baseline VA, even though the CDVA did not have obvious improvement, all the other visual function parameters (OSI, VF-14 score, SR, MTF-cut off, PVA 100%, PVA 20%, PVA9%) were significantly improved. These results indicated that in the early stage of PCO, because of the uneven posterior capsular opacity, light can still pass through the gap of opacity, visual impairment is not apparent. However, light sacttering has increased significantly, and the visual function has been affected. OSI is more sensitive than VA in evaluating the visual function. OSI can quantify the intraocular scattering caused by PCO. It is an objective and effective PCO evaluation method. Especially in the early stage of PCO, OSI can confirm the subjective symptoms of PCO patients and guide the timing of surgical intervention. In previous studies, Artal et al. proposed an OSI-based cataract classification method: OSI less than 1.0 is the normal eye, 1.0 to 3.0 corresponds to early cataract, 3.0 to 7.0 corresponds to advanced cataract, and 7.0 corresponds to mature cataract [39], and OSI ≥ 3 can be used as a reference for cataract surgery. In our study, OSI ≥ 3 in all patients before the capsulotomy and visual function parameters were significantly improved after surgery, which suggested that OSI ≥ 3 for cataract surgery can also be used for laser capsulotomy.

The double-pass system has recently been applied to objectively evaluate crystalline lens opacity [22, 23, 39]. Because ocular scatter increases under cataract conditions, the measurement of scatter is a good tool for evaluating cataracts. The OSI can be useful parameters to discriminate objectively clear crystalline lens from cataract and beneficial in the decision-making process, particularly in patients with minimal visual acuity loss yet symptomatic cataract [23]. The OSI also has a good objective evaluation of posterior capsule opacification [32]. In this study, we found according to the ROC curve analysis, in the precapsulotomy logMAR CDVA > 0.1 group, the AUC of the OSI was 0.996 (*P* = 0.000). In the precapsulotomy logMAR CDVA ≤0.1 group, the AUC of the OSI was 0.943 (*P* = 0.000), suggesting that the OSI had high sensitivity and accuracy of PCO diagnosis. Thus, the OSI is a useful test for decision-making in PCO surgery. The OQAS provide good repeatability and reproducibility of subjective measurement of intraocular scattering in healthy subjects [20, 43, 44]. The measurements of optical quality and intraocular scattering in children by the double-pass system also showed good intro- and intersession repeatability [13]. The repeatability of OQAS-II is good in measuring the visual quality of normal as well as forme fruste keratoconus [45]. Thus, the OSI can quantitatively analyze the extent of PCO and has the characteristics of high accuracy, simple operation, and excellent clinical application.

In this study, only aspherical monofocal IOL was involved. Some recent studies reported that significantly higher values of OSI and lower values of MTF cutoff and SR were found in the multifocal phakic eyes than monofocal IOL eyes and normal elder [46, 47]. The diffractive or refractive rings in the optics of multifocal IOLs may be partially responsible for causing scatter, resulting in moderate glare [46]. Thus, assessing the decision of laser capsulotomy after multifocal IOL implantation may require more other parameters.

## Conclusions

In conclusion, the OSI based on the double-pass system was useful for evaluating posterior capsule opacification and predicting beneficial capsulotomy in monofocal phakic eyes. Especially for patients with slight posterior capsule opacification and better visual acuity, OSI is more valuable than CDVA. Because OSI has a stronger correlation with EPCO score and VF-14 and is a complete objectivity examination. Thus, the OSI based on the double-pass system may be a useful tool for objective PCO evaluation and providing strong evidence for the indication of laser capsulotomy.

## Data Availability

The datasets used and analysed during the current study available from the corresponding author on reasonable request.

## Abbreviations

OQAS: Optical quality analysis system
CDVA: Corrected distance visual acuity
VF-14: Visual function 14 index
OSI: Objective scattering index
PCO: Posterior capsule opacification
EPCO 2000: Evaluation of PCO 2000
Nd:YAG: Neodymium: yttrium-aluminum-garnet
MTF: Modulation transfer function
SR: Strehl ratio
PVA: Predicted visual acuity
TDRS: Early treatment diabetic retinopathy study
